# The development and psychometric evaluation of the Chinese Big Five Personality Inventory-15

**DOI:** 10.1371/journal.pone.0221621

**Published:** 2019-08-27

**Authors:** Xintong Zhang, Meng-Cheng Wang, Lingnan He, Luo Jie, Jiaxin Deng

**Affiliations:** 1 Department of Psychology, Guangzhou University, Guangzhou, China; 2 The Center for Psychometrics and Latent Variable Modeling, Guangzhou University, Guangzhou, China; 3 The Key Laboratory for Juveniles Mental Health and Educational Neuroscience in Guangdong Province, Guangzhou University, Guangzhou, China; 4 School of Communication and Design, Sun Yat-Sen University, Guangzhou, China; 5 School of Education, Guizhou Normal University, Guiyang, China; Universitat zu Lubeck Institut fur Psychologie I, GERMANY

## Abstract

The Chinese Big Five Personality Inventory (CBF-PI), a 134-item self-report scale, and its 40-item brief version (CBF-PI-B) are sound psychometric instruments used to measure the Big Five personality domains in the Chinese population. However, their applicability is limited by their length, as well as restricted by assessment conditions. In this study, we developed and validated a new shortened version with 15 items (CBF-PI-15) through exploratory factor analysis and confirmatory factor analysis in a large sample (Sample 1) of 10,738 Chinese adults (mean = 33.90 years, *SD* = 9.39 years, range 17–57 years). Measurement invariance results suggested the CBF-PI-15 were invariant across gender and age groups. Convergent, discriminant and criterion validities were tested in Sample 2 (*N* = 256, mean = 21.62 years, *SD* = 3.06 years, range 18–35 years) and findings showed an expected correlational pattern with external variables. Results revealed positive correlations of Neuroticism with the Barratt Impulsiveness Scale Brief Version (BIS-Brief), the Patient Health Questionnaire, and the Generalized Anxiety Disorder Screener, as well as a strongly negative correlation between Conscientiousness and BIS-Brief. Additionally, Conscientiousness positively correlated with academic performance as expected. In conclusion, the CBF-PI-15 holds promise as an informative alternative for the original CBF-PI-B when administration time or conditions are limited, and our findings provide preliminary support for the utility of the CBF-PI-15.

## Introduction

The Big Five model of personality traits is one of the well-established paradigms for the conceptualization of human personality [1, 2]. However, it is not a comprehensive taxonomy of natural language personality descriptors, and excludes assessable terms and state descriptors [3]. Admittedly, the Big Five has been advocated for as a basic framework for personality description and assessment due to its replicability and ubiquity [4] across numerous cultures over the years. Generally, Extraversion (E), Agreeableness (A), Conscientiousness (C), Neuroticism (N; emotional stability), and Openness to experience (O), each having several facets in a hierarchical structure, are widely accepted as five relatively independent factors which account for phenotypic personality variations between people. The Big Five factor framework has profoundly affected studies about individual personality differences [1], and to date, an extensive body of evidence has corroborated close associations of personality traits with extant variables such as academic performance, impulsiveness, and depression (e.g., [5–7]).

The wide acceptance of the Big Five framework by personality researchers highlights a need for an efficient, reliable, and valid instrument to measure these traits. The 240-item Revised NEO Personality Inventory (NEO-PI-R; [8]) with six facets per dimension, and the NEO Five-Factor Inventory (NEO-FFI; [8]), a brief version of the NEO-PI-R with 60 items, are two of the most commonly utilized questionnaires to assess personality using the Big Five model. However, these two measures are proprietary instruments, which inhibits their availability for research use [9, 10]. Alternatively, the 44-item Big Five Inventory (BFI-44; [11]) was constructed in the late 1980s as a creatively sufficient personality inventory for non-commercial use. A total of 44 short-phrase items were developed, to be responded to in five minutes or less [12], greatly shortening the assessment time.

Although the NEO-PI-R, NEO-FFI, and BFI-44 continue to be widely used, there has been a recent tendency towards developing shorter measures [13]. Shortened personality questionnaire assessments often have psychometric limitations in certain aspects (e.g., reliability or content validity) as expounded upon by Credé and co-investigators (2012) [14], but show attractive benefits when considering the balance between practice and psychometric properties. Specifically, the shortened forms minimize assessment time, avoiding problems of respondents feeling fatigued and bored, meaning that respondents rate each item with more intention and focus which will then lead to more accurate responses [15]. As such, negative participant reactions (i.e., refusal to respond, careless or random responses due to fatigue) can be avoided to some extent by using the shortened forms [14]. More importantly, the shortened forms retain the original measures’ conceptual focus, reliability, and validity, and have a good convergence with the corresponding facets of the original form (e.g., [16–18]).

Examples of this trend of minimal measurements included the 10-item Big Five Inventory (BFI-10; [12]) and the 20-item Mini-IPIP [19]. For instance, the BFI-10 demonstrates good convergent validity with the NEO-PI-R, good external validity with peer ratings, test-retest reliability, and comparative or even improved predictive validity against the BFI-44 [10, 12]. Additionally, Donnellan and colleagues (2006) [19] designed the Mini-IPIP on the basis of two considerations (i.e., the aim to maintain content coverage and acceptable psychometric properties, and the desire to evaluate each personality domain with fewer items), and demonstrated favorable results (e.g., excellent content coverage, high test–retest correlations) using a large Western undergraduate sample. Likewise, Baldasaro and colleagues (2013) [20] replicated the previous findings and suggested that the Mini-IPIP had acceptable reliability, partial or full metric invariance, and exhibited some degree of criterion validity when drawing on a representative sample of American young adults. More recently, two short German forms of the Next Big Five Inventory (BFI-2; [21]), namely the 30-item BFI-2-S and the 15-item BFI-2-XS, have been developed by Soto and John (2017b) [22] to address the common limitations that the original long version might possess in some circumstances, such as being a large-scale survey or for when participants need to complete the same personality measure multiple times. As expected, both the BFI-2-S and BFI-2-XS maintained much of the full measure’s reliability and validity at the level of the Big Five domains, and the BFI-2-S has been proven to be useful when assessing facet personality traits among reasonably large populations [22]. Overall, the quality of psychometric properties of the relevant long or shortened forms measuring the Big Five personality structure have been robustly validated for Western populations [10].

From a cross-cultural perspective, it is important to replicate the Big Five model in a non-Western sample (e.g., Chinese). At present, questionnaire assessments for the Big Five model have been translated and revised, and are being used widely in China. McCrae and colleagues (1996) [23] translated the NEO-PI-R into Chinese, and yielded strong evidence of the cross-cultural replicability of the NEO-PI-R factor structure and the Big Five model in a sample of 352 Hong Kong college students. Subsequently, Dai and colleagues (2004) [24] further confirmed the factor structure and psychometric properties of the revised Chinese version of the NEO-PI-R in Mainland China community adults. Similarly, those findings were also replicated using Chinese college students using the Chinese version of the NEO-FFI [25], and Zheng and colleagues (2008) [26] confirmed the five-factor model when using the Chinese-language IPIP Big Five factor markers. Despite these promising findings, however, there are still a number of limitations in existing research. Specifically, Zheng et al. (2008) [26] evaluated the BFI-44 using Chinese participants, but did not report the details about the resource translation or psychometric properties. Leung and colleagues (2013) [27] translated the BFI-44 into traditional Chinese characters to conduct research with Hong Kong-based participants, but it was difficult to use the same questionnaire widely in Mainland China where people generally use simplified characters. Notably, a previous meta-analysis study [28] has demonstrated that the Cronbach’s αs in Chinese samples were lower than those in the foreign samples when using the same Western Big Five personality inventories. Also, the reliability coefficients of the revised Western Big Five factor personality measures were lower than the self-developed Chinese Big Five factor personality inventory that was applied in China [28]. Moreover, the limitation on the length of the aforementioned measures should be taken into consideration.

To overcome these existing limitations of the Big Five personality measures, Wang and colleagues (2010a; 2010b) [29, 30] developed the Chinese Big Five Personality Inventory (CBF-PI). The CBF-PI consists of 134 items with 22 facets, and participants respond to each item based on a six-point Likert scale that ranges from 1 (“disagree strongly”) to 6 (“agree strongly”). Results indicated the CBF-PI had favorable internal consistency (Cronbach’s α), ranging from .83 (A) to .91 (C), and the test-retest reliability coefficients were above .80 [29]. Additionally, the CBF-PI correlated strongly with relevant dimensions of the NEO-PI-R and BFI, ranging from .45 (A) to .62 (E), and .69 (A) to .82 (O), respectively [30]. An abbreviated measure, the CBF-PI-B, was developed from the CBF-PI. Taking the total score of each dimension as a marker variable, the criteria of selecting adequate items to report on were as follows: (a) the correlation of the item with the marker variable was > .40; (b) the factor loading of the item on the non-target dimension was < .40; (c) the selected items must have come from different facets (i.e., the resulting items should cover multiple content domains). If the selected item did not meet the above criteria, it was replaced until every item satisfied the criteria. Through this process, a total of 40 items were chosen to form the shortened version [31]. Similar to the original version, the CBF-PI-B demonstrated excellent Cronbach’s α coefficients (ranging from .76 [A] to .81 [N]), good test-retest reliability (ranging from .67 [A] to .81 [N]), and its five dimensions correlated robustly with the corresponding dimensions of both the NEO-PI-R (*r* = .36 [A] to .85 [C]) and BFI-44 (*r* = .58 [A] to .83 [N]). Equivalence coefficients between the CBF-PI-B and the original being above .85, the shortened version was regarded as a promising self-report tool that would be a good substitute for the original form to assess the Big Five personality traits in Chinese samples. Overall, both the CBF-PI and the CBF-PI-B dovetail with Chinese idiomatic expressions, and have since become widely applied to various Chinese groups (e.g., [32, 33]). Still, concerns regarding the length of the CBF-PI and the CBF-PI-B should still be taken into consideration.

### Current study

The primary purpose of the present study was to develop and evaluate the psychometric properties of a more time-effective form of the CBF-PI-B. First, we validated the original CBF-PI-B on a sample of participants, and then used these results to create a more simplified 15-item version of the CBF-PI-B (CBF-PI-15). The factor structure was then tested through confirmatory factor analysis (CFA). Fifteen items were expected to provide adequate coverage of the five factors, as well as excellent psychometric properties, while showing a meaningful reduction in response time, particularly in an epidemiological survey. Second, measurement invariance analyses were then performed to examine whether the factor structure of the CBF-PI-15 was equivalent across gender and age groups by testing configural, metric, scalar, and error invariances. Third, the convergent and discriminant validity of the CBF-PI-15 was examined by correlating the CBF-PI-15 subscale scores to the subscale scores of alternative self-report measures that identify personality traits and other relevant assessments that measure impulsiveness, depression, and anxiety in a new sample. The correlations with the original CBF-PI-B were also calculated. Finally, the criterion validity of the CBF-PI-15 was scrutinized by correlating the CBF-PI-B five factor scores to academic performance, the relevant external variable. We also reported the internal consistency, including the Cronbach’s α and mean inter-item correlations, of the CBF-PI-15. We expected the abbreviated inventory to hold promise as an informative alternative for the original CBF-PI-B, showing favorable psychometric properties.

## Materials and methods

### Participants

Two independent samples were used. The first sample included 11,218 adult participants (62.2% male, 37.8% female) who had completed the CBF-PI-B online in September 2017. Most participants (93.9%) were residents of Mainland China, with a smaller percentage of them (6.1%) living abroad. Considering most current studies using the CBF-PI-B intend to measure adult personality traits, we excluded data from those under 17 years of age. Finally, a total of 10,738 participants (62.4% males; mean = 33.90 years; *SD* = 9.39 years; range 17–57 years) were included in the first sample. The second sample of 256 college students (mean = 21.62 years; *SD* = 3.06 years; range 18–35 years) was recruited anonymously from Guangzhou University in China for partial fulfillment of a course requirement. The gender breakdown was 32.0% male and 67.2% female, with two participants not reporting their gender. Regarding ethnic origin, 96.5% of participants were Han, the majority ethnic group in China, and only four students were from other ethnic groups.

This study was approved by the Human Subjects Review Committee at Guangzhou University. All questionnaires were administered to those who had given informed consent.

### Measures

#### The Chinese Big Five Personality Inventory Brief Version (CBF-PI-B)

The CBF-PI-B [31], an abbreviated form of the CBF-PI [29, 30], was developed to evaluate the five dimensions of the Big Five personality model. The CBF-PI-B is administered as a self-report measure consisting of 40 items. Items 5, 8, 13, 15, 18, 32, and 36 are scored reversely. Each item is answered on a six-point Likert-type scale, ranging from 1 (“disagree strongly”) to 6 (“agree strongly”). The Cronbach’s α coefficients in Sample 1 were .812, .771, .736, .765, and .762, for N, C, A, O, and E, respectively. In Sample 2, the Cronbach’s α coefficients were .859, .779, .745, .781, and .780, respectively.

#### The Mini-International Personality Item Pool Five-Factor Model (Mini-IPIP)

The Mini-IPIP [19] is based on the 50-item International Personality Item Pool five-factor model (IPIP-BF; [34]). This instrument contains 20 items that are rated on a five-point Likert-type scale, ranging from 1 (“disagree strongly”) to 5 (“agree strongly”). The Cronbach’s α coefficients for Sample 2 were .712, .598, .609, .707, and .734 for N, C, A, O, and E, respectively.

#### The 10-Item Big Five Inventory (BFI-10)

The BFI-10 [12] is an abbreviated version of the BFI-44 [11]. Its 10 items are short, descriptive phrases that participants rate on a five-point Likert-type scale, ranging from 1 (“disagree strongly”) to 5 (“agree strongly”). The BFI-10 assesses the Big Five model using two items per dimension, one coded to be in a positive direction, and the other in a negative direction on the scale. Previous research has confirmed that the construct validity of the BFI-10 is dependable because it retains an extensive portion of the reliability and validity of the initial BFI-44 [12]. In the current study, Cronbach’s α coefficients in Sample 2 for N, C, A, O, and E were .553, .359, .251, .289, and .549, respectively.

#### The Barratt Impulsiveness Scale Brief Version (BIS-Brief)

The BIS-Brief [35] is a shortened form of the BIS-11 [36]. A total of eight items are rated to assess general impulsivity, with each item scored on a four-point scale, ranging from 1 (“rarely/never”) to 4 (“almost always/always”). The Chinese version of the BIS-Brief was validated in a sample of Chinese male prisoners and showed good reliability and construct validity [37]. The Cronbach’s α of the BIS-Brief in Sample 2 of the present study was .753.

#### The Patient Health Questionnaire-9 (PHQ-9)

The PHQ-9 [38] is a nine-item instrument designed to assess depression, and is based on the DSM–IV diagnostic criteria for major depressive disorder. Each item is rated on a four-point scale, ranging from 1 (“not at all”) to 4 (“nearly every day”). In the current study, the Cronbach’s α coefficient of Sample 2 was .823.

#### The Generalized Anxiety Disorder Screener-7 (GAD-7)

The GAD-7 [39] is a brief self-report scale that uses seven items to identify generalized anxiety in primary care. The items are rated on a four-point scale, ranging from 1 (“not at all”) to 4 (“nearly every day”). The Cronbach’s α coefficient of Sample 2 in this study was .895.

#### Academic performance

Participants reported their academic performance as a comparison to their classmates. Responses were rated from 1 (“at the lower percentile”) to 5 (“at the upper percentile”). The results showed that 6.3% (*N* = 16) were in the lower percentile, 16.8% (*N* = 43) were in the middle-to-lower percentile, 39.5% (*N* = 101) were in the middle percentile, 24.6% (*N* = 63) were in the middle-to-upper percentile, and 12.5% (*N* = 32) were in the upper percentile, with one participant refusing to respond.

### Statistical analyses

First, we examined the factor structure of the CBF-PI-B for Sample 1 with M*plus* 7.4 [40]. The data was divided randomly into two parts: Sample A and Sample B. Each sample included 5,369 participants. Sample A was chosen at random for exploratory factor analysis to develop an abbreviated form. Robust Maximum Likelihood (MLR) with oblique rotation was adopted. Factor loadings less than .30 or more than .30 on more than one factor were dropped [41]. Based on the Big Five model, we selected three items from each dimension whose factor loadings were relatively high but whose cross-loadings were distinctly low to be included in the CBF-PI-15. Following the EFA, Sample B was then used for confirmatory factor analysis. Fit indices, including chi-square (*χ*^2^), the standardized root mean square residual (SRMR), the root mean square error of approximation (RMSEA), the Tucker-Lewis index (TLI), and the comparative fit index (CFI), were computed to assess the goodness of fit of the model. Conventional guidelines suggest a cutoff value close to .08 for SRMR, and a cutoff value close to .06 for RMSEA [42]. Moreover, CFI and TLI values ≥ .90 indicate an adequate model fit [43].

Second, measurement invariance (MI) tests were conducted across gender and age groups of Sample 1 using a series of multi-group CFAs. MI was examined at four levels (configural invariance, metric invariance, scalar invariance, and error variance invariance), and the differences in CFI (ΔCFI) and TLI (ΔTLI) were regarded as suitable indicators of measurement invariance [44]. Additionally, ΔCFIs ≤ .01 and ΔTLIs ≤ .010 indicated that the invariance hypothesis should be accepted, as mean differences exist when ΔCFIs and ΔTLIs are between or equal to .01 and .02, and definite differences exist when ΔCFIs and ΔTLIs are *>* .02 [44]. Based on the taxonomy of the invariance tests, latent mean invariance was then performed across gender and age groups in Sample 1 to detect latent mean differences.

Third, to assess internal consistency of the CBF-PI-15 subscales scores, Cronbach’s α [45] was calculated using SPSS (IBM, SPSS version 19, 2010). Ranges of measures were as follows: < .60 = insufficient; .60 to .69 = marginal; .70 to .79 = acceptable; .80 to .89 = good; and .90 or higher = excellent [46]. However, Cronbach’s α depends on inter-item correlations and number of items. Therefore, mean inter-item correlations (MIC) were also computed to be used as straightforward indicators of the scale’s internal consistency (i.e., not simply an effect of a few particular items), and was considered to be adequate if between .15 and .50 [47].

Finally, to assess the convergent, discriminant and criterion validity of the 15-item version, Pearson correlations of the CBF-PI-15 were performed with SPSS 19.0 using Mini-IPIP, BFI-10, BIS-Brief, PHQ-9, GAD-7, and academic performance. Meanwhile, associations between the CBF-PI-15 subscales and the original CBF-PI-B subscales were computed and given further examination. According to Cohen’s guidelines [48], a correlation (*r*) of ≤ .29 is interpreted as being weak, an *r* from .30 to .49 is interpreted as being moderate, and an *r* ≥ .50 is interpreted as being robust. The hypothesis was that the CBF-PI-15 five subscales would correlate strongly with the corresponding subscales of the original and alternative personality measures, and that the CBF-PI-15 would also exhibit significant associations with relevant external variables (i.e., impulsiveness, depression, anxiety, and academic performance), in line with previous findings (e.g., [5–7]). Finally, to determine whether the strength of the correlation of the abbreviated CBF-PI-15 with the criterion tools differed from that with the original CBF-PI-B or the other personality assessments, we employed the method proposed by Dunn and Clark (1969) [49] (c.f., [50]) using a spreadsheet that was developed by DeCoster and Lselin (2005) [51] and that can be retrieved at: http://stat-help.com/spreadsheets.html.

## Results

### Preliminary analyses

Descriptive statistics, including Cronbach’s αs, means, standard deviations, and number of items about all relative measures in the current study are presented in Table 1.

**Table 1:** Descriptive statistics for all scales in the current study.

		α	MIC	Mean	*SD*	*N* of items
	CBF-PI-B					40
Sample 1 (*N* = 10,738)	N	.812	.349	3.45	1.06	8
	C	.771	.298	4.23	0.87	8
	A	.736	.260	4.34	0.84	8
	O	.765	.289	3.89	0.90	8
	E	.762	.284	3.51	0.95	8
	CBF-PI-15					15
	N	.747	.496	3.57	1.33	3
	C	.611	.344	4.26	1.07	3
	A	.740	.487	4.29	1.16	3
	O	.803	.577	3.01	1.31	3
	E	.738	.484	3.06	1.30	3
Sample 2 (*N* = 256)	CBF-PI-B					40
	N	.859	.435	3.19	0.87	8
	C	.779	.308	4.14	0.66	8
	A	.745	.270	4.34	0.62	8
	O	.781	.309	4.01	0.67	8
	E	.780	.308	3.66	0.76	8
	CBF-PI-15					15
	N	.809	.584	3.28	1.02	3
	C	.612	.347	4.03	0.81	3
	A	.769	.527	4.39	0.85	3
	O	.811	.589	3.44	1.00	3
	E	.721	.463	3.54	1.00	3
	Mini-IPIP					20
	N	.712	.378	3.10	0.75	4
	C	.598	.278	3.56	0.64	4
	A	.609	.283	3.67	0.56	4
	O	.707	.376	3.48	0.68	4
	E	.734	.411	2.89	0.78	4
	BFI-10					10
	N	.553	.383	3.18	0.75	2
	C	.359	.224	3.26	0.69	2
	A	.251	.145	3.78	0.62	2
	O	.289	.174	3.69	0.75	2
	E	.549	.378	3.28	0.83	2
	BIS-Brief TOT	.753	.275	2.20	0.38	8
	PHQ-9 TOT	.823	.351	1.81	0.43	9
	GAD-7 TOT	.895	.550	1.82	0.55	7

*Note*. N = Neuroticism; C = Conscientiousness; A = Agreeableness; O = Openness; E = Extraversion; CBF-PI-B = Chinese Big Five Personality Inventory Brief Version; CBF-PI-15 = Chinese Big Five Personality Inventory Brief 15-item Version; Mini-IPIP = Mini-International Personality Item Pool five-factor model; BFI-10 = Big Five Inventory 10-item version; BIS-Brief TOT = Barratt Impulsiveness Scale Brief Version total score; PHQ-9 TOT = Patient Health Questionnaire total score; GAD-7 TOT = Generalized Anxiety Disorder Screener total score; α = Cronbach’s alpha; MIC = mean inter-item correlation; *SD* = standard deviation.

### Development of the CBF-PI-15

Developing a brief, psychometrically strong personality inventory based on the five-factor model for wide use within the Chinese population was the central purpose of abbreviating the CBF-PI-B. Item reduction was achieved using Sample A through a stepwise selection process with EFA. Given the aforementioned conventional guidelines and the particular five factors, those items with loadings below .30 or higher than .30 on more than one factor were eliminated. A total of 15 items were selected to construct the shortened CBF-PI-15 by identifying the three highest-loading items from each supported factor. We then experimented with different combinations of these 15 items for the CBF-PI-15, taking all indicators (e.g., Cronbach’s α coefficients) into consideration. As a result, the CBF-PI-15 was formed using items 21, 26, and 31 to measure N, items 17, 22, and 37 to measure C, items 3, 23, and 33 to measure A, items 9, 14, and 24 to measure O, and items 5, 15, and 35 to measure E. Items 5 and 15 were scored reversely. Of note, as Table 2 demonstrates, all factor loadings of the selected items were higher than .50.

**Table 2:** Factor loading of CBF-PI-15 in Sample 1.

	N	C	A	O	E
21. I often worry about trifles.	.662/.647				
26. I often feel disturbed.	.717/.679				
31. I always worry that something bad is going to happen.	.695/.753				
17. I like to plan things from the beginning.		.576/.528			
22. I am diligent in my work or study.		.518/.488			
37. One of my characteristics is doing things logically and orderly.		.582/.726			
3. I think most people are well-intentioned.			.572/.698		
23. Although there are some frauds in the society, I think most people can be trusted.			.610/.693		
33. Although there are some bad things in human society (such as war, evil, and fraud), I still believe that human nature is generally good.			.657/.749		
9. I'm a person who loves to take risks and break the rules.				.737/.749	
14. I like adventure.				.782/.740	
24. I have a spirit of adventure that no one else has.				.768/.769	
5. I'm bored by parties with lots of people. (R)					.616/.703
15. I try to avoid parties with lots of people and noisy environments. (R)					.647/.792
35. I like to go to social and recreational parties.					.627/.646

*Note*. N = Neuroticism; C = Conscientiousness; A = Agreeableness; O = Openness; E = Extraversion; R = negatively-worded items reverse-scored prior to analysis.

Before virgule is the factor loading of the CBF-PI-15 selected from the results of the EFA model of the CBF-PI-B.

After virgule is the factor loading of the CBF-PI-15 in the CFA model.

All factor loadings are significant at a 5% level.

Sequentially, the factor structure of the CBF-PI-15 was re-examined using Sample B. Fit indices were good for the CBF-PI-15 in the current study (MLRχ^2^ = 918.882, *df* = 80, CFI = .946, TLI = .929, RMSEA = .044, SRMR = .040), supporting the factorial validity of the abbreviated scale.

### Invariance across gender and age groups

Tests of measurement invariance were conducted in Sample 1 to systematically investigate the extent to which the measurement model was replicated across gender and age groups, and to investigate possible latent differences across these subgroups of participants. The results are shown in Table 3. Gender is divided into two groups: male (*N* = 6,698) and female (*N* = 4,040). Age is divided into four groups, 17–20 (*N* = 611; Group 1), 21–29 (*N* = 3,391; Group 2), 30–39 (*N* = 3,772; Group 3), and 40 and older (*N* = 2,964; Group 4). CFIs and TLIs of these four types of measurement invariance varied in gender groups from .923 to .944, and in age groups from .904 to .918. All ΔCFI and ΔTLI were below or equal to .01 among the four levels of measurement invariance, indicating that there was no significant difference in gender groups or age groups [44], and that the CBF-PI-15 model with cross-group equality constraints was the best fit to the data.

**Table 3:** Goodness-of-fit indices and model comparisons for CBF-PI-15 measurement invariance models.

Subgroup	Model	MLRχ^2^	*df*	TLI	CFI	RMSEA (90% CI)		Δχ^2^	Δ*df*	ΔTLI	ΔCFI
Age	A–configural invariance	3039.126***	410	.904	.918	.049 (.047 .050)					
	B–metric invariance	3071.725***	425	.907	.918	.048 (.047 .050)	B vs. A	36.008**	15	+.003	.000
	C–scalar invariance	3172.166***	440	.907	.915	.048 (.047 .050)	C vs. B	99.310***	15	.000	-.003
	D–error variance invariance	3292.432***	485	.914	.913	.046 (.045 .048)	D vs. C	143.263***	45	+.007	-.002
Gender	A–configural invariance	1878.590***	160	.927	.944	.045 (.043 .047)					
	B–metric invariance	1897.817***	170	.931	.944	.044 (.042 .045)	B vs. A	20.931*	10	+.004	.000
	C–scalar invariance	2207.454***	180	.923	.934	.046 (.044 .048)	C vs. B	342.584***	10	-.008	-.010
	D–error variance invariance	2311.641***	195	.926	.931	.045 (.043 .047)	D vs. C	112.089***	15	+.003	-.003

*Note*. MLR = robust maximum likelihood; *df =* degrees of freedom; TLI = Tucker-Lewis index; CFI = comparative fit index; RMSEA (90% CI) = root mean square error of approximation (90% confidence interval).

***p < .001.

** *p* < .01.

* *p* < .05.

**Fig 1:**
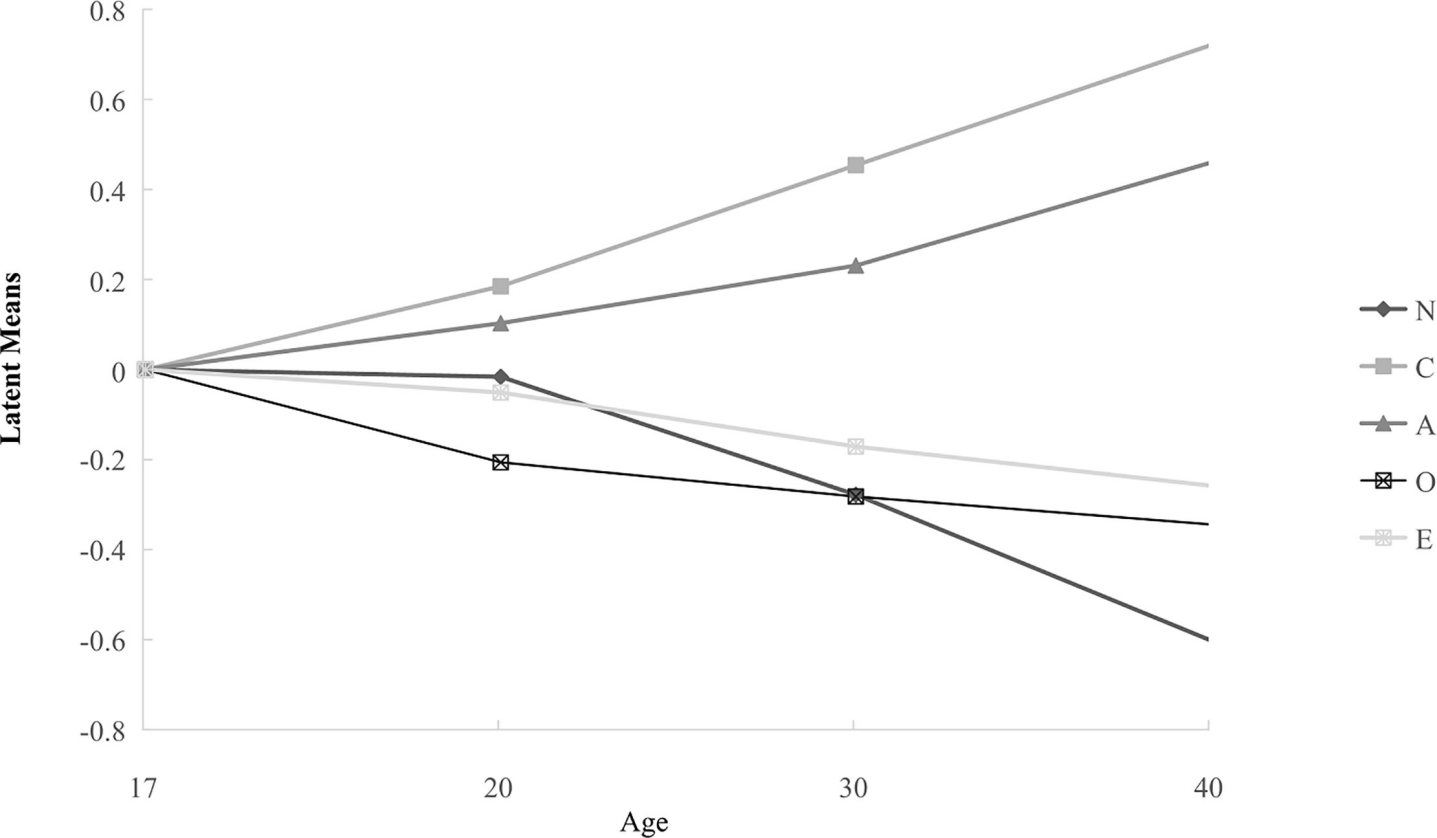
Latent means of Big Five dimensions results across different age groups.

**Fig 2:**
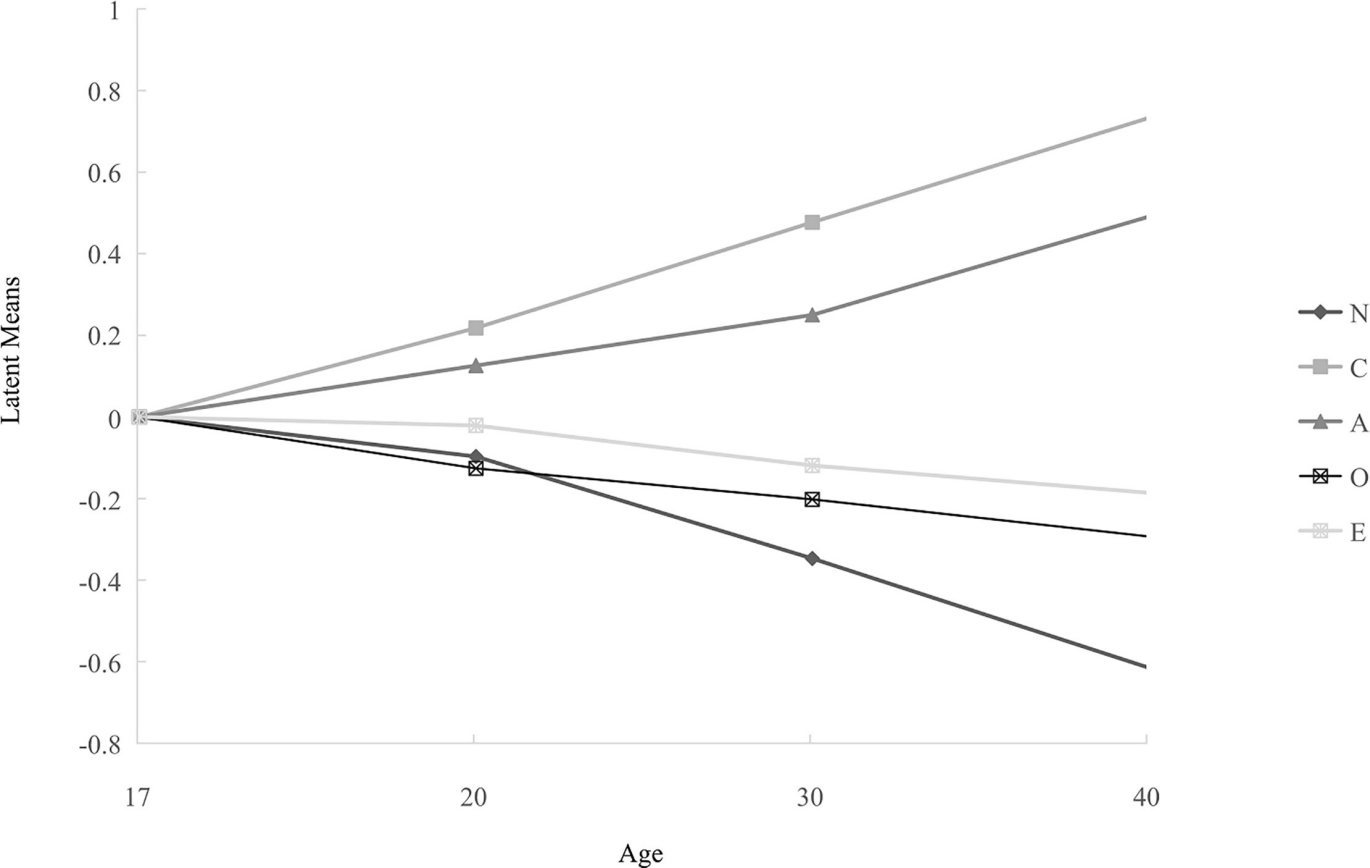
Latent means of males’ Big Five dimensions results across different age groups.

**Fig 3:**
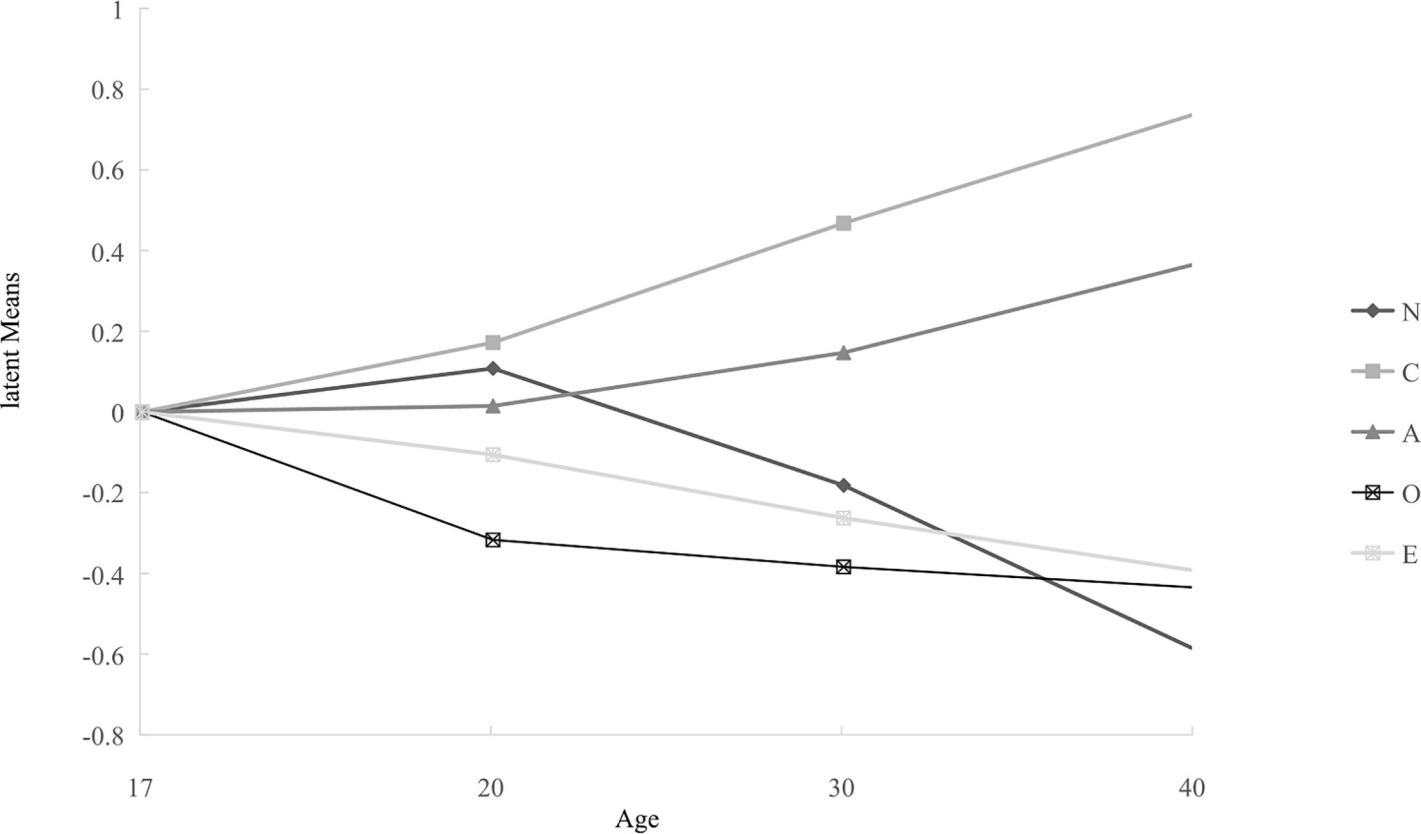
Latent means of females’ Big Five dimensions results across different age groups.

Based on the taxonomy of invariance tests, latent mean differences in gender and age groups were pursued in the current study. Above all, when the latent means of males were fixed to zero for identification purposes, the latent means of females were significantly higher (*p* ≤ .001) for N (.199) and A (.153), but lower for C (-.267), O (-.461), and E (-.103). Additionally, Fig 1 shows the latent means of the five dimensions across age groups and Group 1, the designated reference group. Specifically, the latent means of the older groups, excluding Group 2 (-.016), were significantly lower for N (Group 3 = -.278, Group 4 = -.602), whereas they were significantly higher for C (Group 2 = .185, Group 3 = .454, Group 4 = .721). The older-aged groups had higher latent means for A (Group 2 = .103, Group 3 = .231, Group 4 = .460), and lower latent means for O (Group 2 = -.206, Group 3 = -.282, Group 4 = -.344) when compared to Group 1. With regards to E, Group 1 showed significant but relatively weak differences in latent means in comparison to the other four groups (Group 2 = -.051, Group 3 = -.171, Group 4 = -.258). Meanwhile, Figs 2 and 3 show the results of invariance across all 10 groups, representing all combinations of the two genders and five age categories (age 17–20 males, age 17–20 females, age 21–29 males, age 21–29 females, etc.).

### Internal consistency

The internal consistency of the two samples of the CBF-PI-15 is shown in Table 1. The internal consistency of the CBF-PI-15 factor scores ranged from moderate to good. Namely, Cronbach’s αs ranged from .611 (C) to .803 (O), and all MICs except for the O factor were in the range of .15 to .50 in Sample 1. In Sample 2, the internal consistency of the CBF-PI-15 was acceptable in both Cronbach’s α and MIC, although the internal consistency of the A, N, and O factor scores was only favorable in Cronbach’s α (A = .769, N = .809, O = .811), but not in MIC (MICs > .50).

### Convergent and discriminant validity

Table 4 shows the correlations between the CBF-PI-15 and the alternative personality measures. Results demonstrate favorable associations of the CBF-PI-15 with the original CBF-PI-B. The corresponding five dimensions all showed correlations of > .75 (mean = .832), with all discriminant correlations being < .40 (mean = .194). The CBF-PI-15 also showed acceptable convergent correlations with the Mini-IPIP domains (mean = .414, range = .219 to .546), and with the BFI-10 (mean = .439, range = .238 to .590). In contrast, the correlations of the corresponding dimensions between the Mini-IPIP and BFI-10 ranged from .197 to .676 (mean = .516), and the mean discriminant correlation was .208.

**Table 4:** Correlations between the CBF-PI-15, Mini IPIP, BFI-10, and CBF-PI-B.

	CBF-PI-15					Mini-IPIP					BFI-10					CBF-PI-B				
CBF-PI-15	N	C	A	O	E	N	C	A	O	E	N	C	A	O	E	N	C	A	O	E
N	1																			
C	-.187^b^	1																		
A	-.169^b^	.193 ^b^	1																	
O	-.089	.133 ^c^	.057	1																
E	-.263^a^	.057	.178 ^b^	.165 ^b^	1															
Mini-IPIP																				
N	**.546**^a^	-.201 ^a^	-.100	-.184 ^b^	-.190 ^b^	1														
C	-.328^a^	**.503** ^a^	.052	.058	.097	-.322 ^a^	1													
A	-.072	.339 ^a^	**.219** ^a^	.142 ^c^	.309 ^b^	-.020	.320 ^a^	1												
O	-.116	.260 ^a^	-.087	**.262** ^a^	-.014	-.213 ^a^	.273 ^a^	.293 ^a^	1											
E	-.151^c^	.127 ^c^	-.001	.351 ^a^	**.541** ^a^	-.183 ^b^	.183 ^b^	.366 ^a^	.288 ^a^	1										
BFI-10																				
N	**.525** ^a^	-.278 ^a^	-.102	-.289 ^a^	-.268 ^a^	**.594** ^a^	-.362 ^a^	-.143 ^c^	-.229 ^a^	-.282 ^a^	1									
C	-.260 ^a^	**.590** ^a^	.140 ^c^	.055	.080	-.214 ^a^	**.524** ^a^	.331 ^a^	.221 ^a^	.112	-.252 ^a^	1								
A	-.273 ^a^	.143 ^c^	**.407** ^a^	.009	.197 ^b^	-.114	.144 ^c^	**.197** ^b^	-.043	.084	-.163 ^b^	.125 ^c^	1							
O	-.058	.157 ^c^	.026	**.238** ^a^	.074	-.130 ^c^	.220 ^a^	.282 ^a^	**.588** ^a^	.223 ^a^	-.138 ^c^	.093	-.038	1						
E	-.223 ^a^	.089	.068	.396 ^a^	**.435** ^a^	-.259 ^a^	.123	.340 ^a^	.226 ^a^	**.676** ^a^	-.313 ^a^	.135 ^c^	.132 ^c^	.187 ^b^	1					
CBF-PI-B																				
N	**.905** ^a^	-.192 ^b^	-.143 ^c^	-.167 ^b^	-.350 ^a^	.663 ^a^	-.323 ^a^	-.060	-.171 ^b^	-.204 ^a^	.579 ^a^	-.252 ^a^	-.267 ^a^	-.062	-.260 ^a^	1				
C	-.194 ^b^	**.881** ^a^	.247 ^a^	.143 ^c^	.054	-.189 ^b^	.491 ^a^	.357 ^a^	.258 ^a^	.071	-.254 ^a^	.660 ^a^	.154 ^c^	.154 ^c^	.049	-.208 ^a^	1			
A	-.089	.205 ^a^	**.777** ^a^	.082	.254 ^a^	.003	.112	.480 ^a^	.009	.158 ^c^	-.060	.166 ^b^	.380 ^a^	.075	.181 ^b^	-.072	.277 ^a^	1		
O	-.047	.294 ^a^	.235 ^a^	**.794** ^a^	.101	-.186 ^b^	.170 ^b^	.341 ^a^	.487 ^a^	.310 ^a^	-.296 ^a^	.163 ^b^	.074	.441 ^a^	.338 ^a^	-.115	.306 ^a^	.294 ^a^	1	
E	-.196 ^b^	.269 ^a^	.201 ^a^	.420 ^a^	**.805** ^a^	-.195 ^b^	.181 ^b^	.404 ^a^	.161 ^c^	.693 ^a^	-.343 ^a^	.173 ^b^	.167 ^b^	.176 ^b^	.574 ^a^	-.293 ^a^	.269 ^a^	.304 ^a^	.410 ^a^	1

*Note*. N = Neuroticism; C = Conscientiousness; A = Agreeableness; O = Openness; E = Extraversion; CBF-PI-15 = Chinese Big Five Personality Inventory Brief 15-item Version; Mini-IPIP = Mini-International Personality Item Pool five-factor model; BFI-10 = Big Five Inventory 10-item Version; CBF-PI-B = Chinese Big Five Personality Inventory Brief Version

a = *p* < .001

b = *p* < .01

c = *p* < .05.

Additionally, Table 5 shows the correlations between the CBF-PI-15 and the BIS-Brief, PHQ-9, and GAD-7. As expected, the BIS-Brief and C demonstrate a significantly negative correlation (*r* = -.646, *p* < .001), and there is a modestly but significantly positive correlation between the BIS-Brief and N (*r* = .303, *p* < .001). The negative correlation coefficients between the BIS-Brief and A, O, and E ranged from -.035 to -.195. The PHQ-9 exhibited a robustly positive relationship with N (*r* = .500, *p* < .001), and negative correlations with C, E, A, and O (*r* = -.242, *p* < .001; *r* = -.252, *p* < .001; *r* = -.181, *p* < .01; and *r* = -.078, *p* > .05, respectively). The GAD-7 also showed a strongly positive relationship with N (*r* = .566, *p* < .001) and linked negatively to other the four dimensions (*r* = -.106 to -.257).

### Criterion validity

From the results presented in Table 5, academic performance appears to have a relationship with the Big Five personality traits of the CBF-PI-15 (*r* = .068 to .332), and the correlations between academic performance and C as well as N were significant (*r* = .332, *p* < .001; *r* = -.129, *p* < .05, respectively). There were also non-significant or trivial relationships of academic performance with A (*r* = .068), O (*r* = .106), and E (*r* = .109). Table 5 also demonstrates correlations between the alternative personality measures (i.e., original CBF-PI-B, Mini-IPIP, and BFI-10) and external criterion variables. Additional analyses revealed that about three quarters of Pearson correlation coefficients were non-significant (see *z*-tests in Table 5) between the CBF-PI-15 and the CBF-PI-B, as well as between the CBF-PI-15 and the other alternative personality measures, suggesting that the CBF-PI-15 has comparable criterion-related validity with the corresponding measures.

**Table 5:** Correlations between personality measures and criterion-related variables.

		AP	*z*-test score			BIS-Brief	*z*-test score			PHQ-9	*z*-test score			GAD-7	*z*-test score		
			*z*1	*z*2	*z*3		*z*1	*z*2	*z*3		*z*1	*z*2	*z*3		*z*1	*z*2	*z*3
N	CBF-PI-15	-.129^c^	2.782^b^	.236	1.432	.303^a^	.230	.406	-.788	.500^a^	-3.625^a^	.040	1.225	.566^a^	-3.915^a^	-.412	.661
	CBF-PI-B	-.204^a^				.297^a^				.582^a^				.649^a^			
	Mini-IPIP	-.143^c^				.280^a^				.498^a^				.585^a^			
	BFI-10	-.215^a^				.348^a^				.436^a^				.534^a^			
C	CBF-PI-15	.332^a^	-1.471	1.933	1.137	-.646^a^	1.428	-2.731^b^	-.755	-.242^a^	1.117	1.295	1.442	-.128^c^	.560	1.449	1.125
	CBF-PI-B	.374^a^				-.678^a^				-.275^a^				-.145^c^			
	Mini-IPIP	.217^a^				-.517^a^				-.319^a^				-.217^a^			
	BFI-10	.271^a^				-.615^a^				-.320^a^				-.191^b^			
A	CBF-PI-15	.068	-1.703	-2.117^c^	.307	-.035	2.107^c^	5.137^a^	1.440	-.181^b^	.121	.630	.676	-.152^c^	-1.394	-1.003	1.842
	CBF-PI-B	.139^c^				-.123				-.186^b^				-.094			
	Mini-IPIP	.231^a^				-.414^a^				-.229^a^				-.074			
	BFI-10	.047				-.133^c^				-.226^a^				-.274^a^			
O	CBF-PI-15	.106	-2.085^c^	-1.538	.039	-.107	3.703^a^	2.832^b^	1.220	-.078	2.836^b^	2.528^c^	1.134	-.106	.274	.970	-.091
	CBF-PI-B	.189^b^				-.253^a^				-.191^b^				-.117			
	Mini-IPIP	.221^a^				-.315^a^				-.266^a^				-.179^b^			
	BFI-10	.103				-.200^b^				-.165^b^				-.099			
E	CBF-PI-15	.109	-2.852^b^	-1.879	-1.493	-.195^b^	2.697^b^	-.204	-.046	-.252^a^	.291	-1.300	.251	-.257^a^	-1.575	-2.605^b^	.488
	CBF-PI-B	.219^a^				-.297^a^				-.263^a^				-.197^b^			
	Mini-IPIP	.220^a^				-.183^b^				-.176^b^				-.104			
	BFI-10	.207^a^				-.192^b^				-.268^a^				-.288^a^			

*Note*. N = Neuroticism; C = Conscientiousness; A = Agreeableness; O = Openness; E = Extraversion; CBF-PI-15 = Chinese Big Five Personality Inventory Brief 15-item version; CBF-PI-B = Chinese Big Five Personality Inventory Brief Version; Mini-IPIP = Mini-International Personality Item Pool five-factor model; BFI-10 = Big Five Inventory 10-item version; AP = academic performance; BIS-Brief = Barratt Impulsiveness Scale Brief Version; PHQ-9 = Patient Health Questionnaire-9; GAD-7 = Generalized Anxiety Disorder Screener-7; *z*1 = *z*-test score of CBF-PI-15 versus CBF-PI-B; *z*2 = *z*-test score of CBF-PI-15 versus Mini-IPIP; *z*3 = *z*-test score of CBF-PI-15 versus BFI-10

a = *p* < .001

b = *p* < .01

c = *p* < .05.

## Discussion

The primary purpose of the present study was to develop and validate an abbreviated form of the CBF-PI-B. Items for a newly developed CBF-PI-15 were selected by balancing concerns for factor structure, internal consistency, and content representativeness. MI tests indicated that the CBF-PI-15 was invariant across gender- and age-differentiated subgroups. Reliability analysis indicated adequate internal consistency with the full CBF-PI-B, and moreover, expected correlation patterns with the shortened form were found with external variables. In general, the CBF-PI-15 can act as an efficient, reliable, and valid assessment of personality traits that can be administered quickly with minimal administration burden while retaining the reliability and validity of the full CBF-PI-B.

Short-form measures of the Big Five Personality traits (e.g., the BFI-10, BFI-2-XS, Mini-IPIP) have been developed and become widely used by researchers over the years, which could derive from their economic value and sound practical results. First, these shorter personality inventories ensure the accuracy of the external validity of research findings to a reliable extent, in that they take less time to complete, thus effectively lessening the likelihood of negative or erroneous participant responses caused by feelings of boredom or fatigue (e.g., refusal to participate in study, careless responses) [14, 15]. Second, the brevity of the shorter measures also plays an important role in the research setting where there is a need to assess a vast amount of related constructs in addition to personality traits within a limited period time. Third, shorter personality inventories improve the accuracy of face validity due to the ease of item distribution (e.g., lack redundant repetitive items [52]) allowing respondents to better understand the content of each item. Finally, the shorter inventories appear to not correspond with substantial psychometric sacrifices, as Credé and colleagues (2012) [14] have pointed out, and have been validated with comparable psychometric properties such as criterion validity and test-retest reliability when compared with longer scales in previous research [16–18].

However, there are also several notable psychometric concerns about the reliability and validity of dramatically shortened scales. Credé et al. (2012) [14] noted that the shortened tools appear to be limited by measurement error, which relates to reliability and could therefore increase the risk of criterion invalidity. While adding more items could cancel out the risk of measurement error from a psychometric perspective, the quality of information could decrease if informants are asked to respond to numerous seemingly-repetitive questions, which could also have an inverse impact on the reliability, from cognitive perspective [53]. Another concern about shorter personality measures is the risk that it might prioritize one personality facet over the other facets, rather than covering all facets equally. In this case, the shorter measures could lack substantial content as a consequence of including fewer items, suggesting that construct underrepresentation could result in poor predictive validity [54]. Credé and colleagues (2012) [14] also criticized the use of briefer scales when measuring the Big Five as weak associations of Big Five measures with either the criteria or new predictors will substantially increase both Type 1 and Type 2 error rates. Ultimately, shorter measures are recommended when assessing personality traits only if time and space are limited. Otherwise, it is recommended to use the full-length, well-established measurement instruments [12, 19].

Admittedly, the development of short measures in existing research has typically emerged out of practical necessity. In the current study, the search for a brief self-report based on the five-factor model arose from investigating personality traits of Chinese people. The CBF-PI-B has been difficult to use as often as researchers would like because it is too long to include in already-crowded assessment batteries during short time intervals, or it is unsuitable for use due to its intensive and repetitive nature. Therefore, the factor structure of the CBF-PI-B was reinvestigated using EFA in half of Sample 1, and a total of 15 items with high factor loadings and low cross-loadings were used to construct a new abbreviated scale. In assessments of the CBF-PI-15, results were encouraging. First, the CFA was sequentially conducted with the other half of Sample 1. All fit indices were fully favorable (CFI and TLI > .90, RMSEA < .06, and SRMR < .08), supporting the adequacy of the CBF-PI-15 structure. The 15-item abbreviated scale suitably represented the content and structure of the original CBF-PI-B in a condensed format.

Second, measurement invariance and latent means comparisons were tested across gender and age groups. Prior studies (e.g., [13, 55]) have focused on comparing the scores of the five personality dimensions among different gender or age groups without meeting the requirement of measurement equivalence. In the present study, measurement invariance analysis provided some informative perspectives on the measurement properties of the CBF-PI-15 scores. Results revealed that the CBF-PI-15 had strict measurement invariance (i.e., configural invariance, metric invariance, scalar invariance, and error invariance) across both gender and age groups, suggesting that the CBF-PI-15 scores can be interpreted in the same way for various groups of people.

With a satisfying level of measurement invariance, the latent mean differences across gender and age were examined. In line with clear findings from a previous study [56], women showed higher latent means of N and A than those of men on responses to the Big Five Inventory. Men tended to score higher in the extraversion-related trait as Feingold (1994) [57] found, and differences in O between Chinese males and females were apparent. It is possible that social and economic conditions lead to the differences in measured personality traits between males and females [58]. For age differences, our results are consistent with previous related studies [55, 59], and reveal that A and C show a positive age trend, while E and O show relatively smaller differences when compared to age. N is negatively correlated with age, and there is a curvilinear trend peaking in the 21–29 years of age group, which to some extent mirrors the higher incidence of N-related psychopathology as noted by McCrae, Martin, and Costa (2005) [60]. The maturity principle suggests that as an individual grows older, they tend to mature and become more productive contributors to society rather than focusing only on satisfying their needs for self-actualization, from a humanistic perspective. Accordingly, E and O both appear to decrease after young adulthood [61]. Recent research [56] has also shown clear evidence that the latent means differ systematically across age-gender groups. In the current study, older males demonstrate higher scores for A and C, lower scores for N and O, and showed only the smallest negative difference in E. Females show similar patterns to males in latent mean scores in the five dimensions, except in the case of N and O. Women show lower but more stable levels of O when over the age of 20, whereas the latent means of O shows an obvious decrease from the age of 20. Additionally, a large fluctuation in N appears throughout a woman’s life span, indicating that women in early adulthood are more emotionally unstable, but as a woman’s age increases, the latent means trends lower. Considering the abundant variation in observed gender and age differences across previous research [56], there is a need to examine these differences deeper within the Mainland China context.

Third, the CBF-PI-15 maintains adequate internal consistency, though only in approximately one third of the CBF-PI-B’s total items. Cronbach’s α coefficients of the five subscale scores in both samples were higher than those for the alternative short personality measures (i.e., BFI-10 and Mini-IPIP). It was noteworthy that C ranks lowest and O ranks highest in the current study, which is inconsistent with a general finding in personality research overall. For example, Benet-Martinez and John (1998) [62] suggested that A showed the lowest reliability in both English and Spanish versions of the BFI-44. Likewise, this finding seems to be comparable in the Chinese version of BFI-10 [13] and BFI (29-item; [27]). Gnambs (2014) [63] pointed out that situational differences would be more likely to affect A ratings. In the present study, an adverse finding–namely the reliability of C being lower than A–was obtained, which warrants further investigation. Nunnally and Bernstein (1994) [64] asserted that the purpose of a study determines the choice of the required level of reliability. As such, a cutoff of Cronbach’s α value at .70 or even lower seems appropriate when considering the breadth of the construct and time availability into account [65]. In doing this, all personality domains of the CBF-PI-15 show reliability coefficients at an adequate level.

The MIC is considered to be a more straightforward indicator of a scale’s internal consistency than Cronbach’s α as it minimizes the effects of the number of total items. The MICs of the CBF-PI-15 are higher in contrast to those of the CBF-PI-B, suggesting stronger inter-item correlations in the CBF-PI-15. John and Soto (2007) [66] raised concern that a greater overlap between items is more likely to emerge in a narrow assessment of the Big Five personality types due to items tending to evaluate closely-related patterns of behavior. In order to meet the requirement of acceptable reliability of the CBF-PI-15, the 15 selected items would be highly correlated with each other, meaning that there is a possibility of content overlap. To address this issue, we reviewed the content of every item of the CBF-PI-15, and found that while there was minor overlap of content, it was to a minimal degree. and concluded that the results were within the acceptable range.

The convergent validity of the CBF-PI-15 was supported by significant associations between the scores on the CBF-PI-15 and the scores on the other Big Five personality measures (e.g., CBF-PI-B, BFI-10, and Mini-IPIP). Comparing the personality measures on the basis of the Big Five framework, convergent validity with the domain scales was adequate for N, C, and E, but somewhat lower for O and A, which corresponds with previous research [12] (See Table 4). As each of these scales contain a small number of items, there is inevitably a limit in content coverage, and conceptual differences in definitions of personality between the various measures, rather than the particular item selection, resulted in a lower level of convergence in the O and A dimensions [12].The correlations between the CBF-PI-15 subscale scores and the BFI-10/Mini-IPIP subscale scores were approximately equal to those between the Mini-IPIP and BFI-10 subscale scores, however the correlation between BFI-10-O and Mini-IPIP-O was higher. Overall, the abbreviated version of the CBF-PI-B has kept most of the information of the original long instrument, and showed itself to be superior when compared to the two other short personality measures.

The present study also explored the associations between the CBF-PI-15 scores and impulsion, anxiety, and depression. The BIS-Brief measures impulse levels, and the CBF-PI-15 showed negative correlations with C, A, O, and E, and a positive correlation with N. Mao and colleagues (2018) [7] indicated that greater self-control seemed evident in individuals with higher levels of N and C, or low levels of O of the CBF-PI-B, possibly contributing to the individual exhibiting less impulsivity. Moreover, in line with previous findings that N is linked with anxiety and depression [6, 67], both the PHQ-9 and GAD-7 exhibited negative correlations with C, E, A, and O, with a positive relationship with N. As Watson and colleagues (1988) [68] suggested, neuroticism represents the tendency to experience heightened levels of negative emotionality (e.g., anger, anxiety, depression).

The CBF-PI-15’s criterion validity was also tested using academic performance. Poropat (2009) [69] once employed a meta-analysis to conduct a thorough review of the associations of Big Five personality traits with academic performance, which added credibility to the assertion that academic performance is correlated with A, C, and O. However, the current study resulted in different findings that academic performance showed moderate to robust correlations with the five personality dimensions, but the most strongly significant correlation to academic performance was with C, followed by N, with A showing only a slight correlation with academic performance (See Table 5). In contrast to those with higher scores in C, individuals with high scores in A were more willing to participate in social activities to improve their social desirability, therefore possibly ignoring the importance of knowledge and skills [70]. Alternatively, results of the current study suggest that the Chinese educational style particularly emphasizes independence over co-operative learning, which may also lead to differences in our findings, meaning that students with a higher level of C will show stronger academic performances. As for the negative correlations with N, Uppal and Mishra (2014) [70] also found that individuals with a professional education appeared to have mastered strategies to control emotional reactions, which could be related to their high scores in N or E.

Finally, although the CBF-PI-B seems perform better than the CBF-PI-15 in terms of convergent and criterion validity, additional analysis in the current study revealed that most correlational coefficients were non-significant (see *z*-tests in Table 5). Consequently, the scores for the CBF-PI-15 and the alternative personality assessments (CBF-PI-B, BFI-10, Mini-IPIP), especially the lengthier version of the CBF-PI-B, present the same substantial pattern of correlations with external variables, including impulsivity, anxiety, depression, and academic performance, supporting the validity of the CBF-PI-15.

In general, our results support the idea that the performance of the CBF-PI-15 is comparable to that of the original CBF-PI-B scores when assessing the five personality dimensions in a Chinese cultural context. The CBF-PI-15 also has additional credence for its utility as a shorter personality assessment. However, the findings of this study should be considered in light of its limitations. First, all data in this study was collected through self-reporting instruments, which can result in shared method variance. Thus, future studies should utilize multiple methods of data collection, as well as make use of multiple informants. Second, this was in essence cross-sectional research, and lacked other more psychometric assessments such as test-retest coefficients. Longitudinal studies evaluating correlations over time, as well as test-retest analysis, should be conducted in future research. Finally, more external criterion-related variables (e.g., self-esteem, stress resistance, concentration; [13, 21]) and other variants in the Chinese sample should be used in future research to provide more robust evidence for the broader validation of the abbreviated CBF-PI-15.

## Conclusions

The current study has contributed to the development of the CBF-PI-15, an abbreviated form of the 40-item Chinese Big Five Personality Inventory Brief Version (CBF-PI-B). Results revealed that the CBF-PI-15 has a good factor structure, acceptable internal consistency reliability, and, as expected, convergent, discriminant, and criterion-related validity. Overall, the shortened version of the inventory holds promise as an informative alternative to the original CBF-PI-B, intended for use when there is a limit of time or space available for the research, or where personality is not a main point of study focus. Although researchers (e.g., [12]) will insist on using the full assessment measure to obtain more stable personality results, the CBF-PI-15 enriches the number of brief Chinese personality measurements and offers more variety in available methods to assess the Big Five personality dimensions.

## Supporting information

S1 Table Datasets sample 1. (XLS)

S2 Table Datasets sample 2. (XLS)

S1 File The Chinese Big Five Personality Inventory-15. (DOCX)
