# Supplementary material for: The development and psychometric evaluation of the Chinese Big Five Personality Inventory-15
Source: PLoS One. 2019 Aug 27;14(8):e0221621. doi: 10.1371/journal.pone.0221621 (PMC6771307; doi:10.1371/journal.pone.0221621)
Supplement: S1 File — (DOCX) [file pone.0221621.s003.docx]

**Supporting Information**

Items of CBF-PI-15 in English

| items | Totally Disagree | Mostly Disagree | A little Disagree | A little  Agree | Mostly  Agree | Fully  Agree |
| --- | --- | --- | --- | --- | --- | --- |
| 21. I often worry about trifles. | 1 | 2 | 3 | 4 | 5 | 6 |
| 26. I often feel disturbed. | 1 | 2 | 3 | 4 | 5 | 6 |
| 31. I always worry that something bad is going to happen. | 1 | 2 | 3 | 4 | 5 | 6 |
| 17. I like to plan things from the beginning. | 1 | 2 | 3 | 4 | 5 | 6 |
| 22. I am diligent in my work or study. | 1 | 2 | 3 | 4 | 5 | 6 |
| 37. One of my characteristics is doing things logically and orderly. | 1 | 2 | 3 | 4 | 5 | 6 |
| 3. I think most people are well-intentioned. | 1 | 2 | 3 | 4 | 5 | 6 |
| 23. Although there are some frauds in the society, I think most people can be trusted. | 1 | 2 | 3 | 4 | 5 | 6 |
| 33. Although there are some bad things in human society (such as war, evil and fraud), I still believe that human nature is generally good. | 1 | 2 | 3 | 4 | 5 | 6 |
| 9. I'm a person who loves to take risks and break the rules. | 1 | 2 | 3 | 4 | 5 | 6 |
| 14. I like adventure. | 1 | 2 | 3 | 4 | 5 | 6 |
| 24. I have a spirit of adventure that no one else has. | 1 | 2 | 3 | 4 | 5 | 6 |
| 5. I'm bored by parties with lots of people. (R) | 1 | 2 | 3 | 4 | 5 | 6 |
| 15. I try to avoid parties with lots of people and noisy environments. (R) | 1 | 2 | 3 | 4 | 5 | 6 |
| 35. I like to go to social and recreational parties. | 1 | 2 | 3 | 4 | 5 | 6 |

Note. The number is the

Items of CBF-PI-15 in Chinese

| **题目内容** | **完全**  **不符合** | **大部分**  **不符合** | **有点**  **不符合** | **有点**  **符合** | **大部分**  **符合** | **完全**  **符合** |
| --- | --- | --- | --- | --- | --- | --- |
| 21.我常担忧一些无关紧要的事情。 | 1 | 2 | 3 | 4 | 5 | 6 |
| 26.我常常感到内心不踏实。 | 1 | 2 | 3 | 4 | 5 | 6 |
| 31.我常担心有什么不好的事情要发生。 | 1 | 2 | 3 | 4 | 5 | 6 |
| 17.我喜欢一开头就把事情计划好。 | 1 | 2 | 3 | 4 | 5 | 6 |
| 22.我工作或学习很勤奋。 | 1 | 2 | 3 | 4 | 5 | 6 |
| 37.做事讲究逻辑和条理是我的一个特点。 | 1 | 2 | 3 | 4 | 5 | 6 |
| 3.我觉得大部分人基本上是心怀善意的。 | 1 | 2 | 3 | 4 | 5 | 6 |
| 23．虽然社会上有些骗子，但我觉得大部分人还是可信的。 | 1 | 2 | 3 | 4 | 5 | 6 |
| 33.尽管人类社会存在着一些阴暗的东西（如战争、罪恶、欺诈），我仍然相信人性总的来说是善良的。 | 1 | 2 | 3 | 4 | 5 | 6 |
| 9.我是个勇于冒险，突破常规的人。 | 1 | 2 | 3 | 4 | 5 | 6 |
| 14.我喜欢冒险。 | 1 | 2 | 3 | 4 | 5 | 6 |
| 24.我身上具有别人没有的冒险精神。 | 1 | 2 | 3 | 4 | 5 | 6 |
| 5.我对人多的聚会感到乏味。(R) | 1 | 2 | 3 | 4 | 5 | 6 |
| 15.我尽量避免参加人多的聚会和嘈杂的环境。(R) | 1 | 2 | 3 | 4 | 5 | 6 |
| 35.我喜欢参加社交与娱乐聚会。 | 1 | 2 | 3 | 4 | 5 | 6 |
